# Neutrophil extracellular traps from healthy donors and HIV-1-infected individuals restrict HIV-1 production in macrophages

**DOI:** 10.1038/s41598-020-75357-2

**Published:** 2020-11-11

**Authors:** Andrés Mojoli, Barbara Simonson Gonçalves, Jairo R. Temerozo, Bruno Cister-Alves, Victor Geddes, Alice Herlinger, Renato Santana Aguiar, José Henrique Pilotto, Elvira M. Saraiva, Dumith Chequer Bou-Habib

**Affiliations:** 1 Laboratory on Thymus Research, Oswaldo Cruz Institute/Fiocruz, Av. Brasil, 4365, Manguinhos, Rio de Janeiro, RJ 21040-900 Brazil; 2 Laboratory of Molecular Virology, Universidade Federal do Rio de Janeiro, Rio de Janeiro, Brazil; 3 Department of Genetics, Ecology and Evolution, Institute of Biological Sciences, Universidade Federal de Minas Gerais, Belo Horizonte, Brazil; 4 Laboratory of AIDS and Molecular Immunology, Oswaldo Cruz Institute/Fiocruz, Rio de Janeiro, Brazil; 5 Laboratory of Immunobiology of Leishmaniasis, Department of Immunology, Paulo de Goes Institute of Microbiology, Universidade Federal do Rio de Janeiro, Av. Carlos Chagas Filho 373, Bloco D/D1-44, Ilha do Fundão, Rio de Janeiro, RJ 21941-902 Brazil; 6 National Institute of Science and Technology on Neuroimmunomodulation, Rio de Janeiro, Brazil

**Keywords:** Immunology, Microbiology, Pathogenesis

## Abstract

Neutrophils release extracellular traps (NETs) after interaction with microorganisms and physiological or synthetic products. NETs consist of decondensed chromatin complexed with proteins, some of them with microbicidal properties. Because NETs can modulate the functioning of HIV-1 target cells, we aimed to verify whether they modify HIV-1 replication in macrophages. We found that exposure of HIV-1-infected macrophages to NETs resulted in significant inhibition of viral replication. The NET anti-HIV-1 action was independent of other soluble factors released by the activated neutrophils, but otherwise dependent on the molecular integrity of NETs, since NET-treatment with protease or DNase abolished this effect. NETs induced macrophage production of the anti-HIV-1 β-chemokines Rantes and MIP-1β, and reduced the levels of integrated HIV-1 DNA in the macrophage genome, which may explain the decreased virus production by infected macrophages. Moreover, the residual virions released by NET-treated HIV-1-infected macrophages lost infectivity. In addition, elevated levels of DNA-elastase complexes were detected in the plasma from HIV-1-infected individuals, and neutrophils from these patients released NETs, which also inhibited HIV-1 replication in in vitro infected macrophages. Our results reveal that NETs may function as an innate immunity mechanism able to restrain HIV-1 production in macrophages.

## Introduction

Neutrophil extracellular traps (NETs) are structures composed of decondensed chromatin fibers complexed with granular and cytoplasmic proteins released by neutrophils after activation by a range of products, including inflammatory mediators, microorganisms, pathogen-associated molecular patterns (PAMPS), and synthetic molecules^1–6^. NETs have been shown to play an important role in the control of infectious diseases. In fact, these structures inactivate and restrict pathogen dissemination through NET-associated proteins endowed with microbicidal properties, including histones, neutrophil elastase, myeloperoxidase, calprotectin and defensins^1,2,7,8^. In contrast, some authors have reported that NETs are central components of the exacerbated inflammatory reaction occurring in individuals with Covid-19, showing that the virus SARS-CoV-2 induces NET formation and that NETs are present in the plasma and lungs of critically ill Covid-19 patients^9–12^.

It has been shown that NET components are able to modulate the phenotype of immune cells^13–16^ and the antimicrobial activities of macrophages^17–21^. Also, human macrophages internalize NETs^22^ and are regulated by these structures, changing their production profile of inflammatory mediators^13,23^. Therefore, NETs can be considered as multifunctional agents, acting both in the direct control of pathogens, through their antimicrobial properties, and in the regulation of the immune responses, after interaction with lymphocytes and macrophages in the tissue microenvironment.

The inflammatory response is an important component of the pathogenesis of the human immunodeficiency virus type-1 (HIV-1) infection, contributing to CD4^+^ T cell death, tissue damage and, thus, to disease progression^24,25^. The HIV-1 infection and subsequent viral replication in the gut-associated lymphoid tissue (GALT) promote tissue injury, neutrophil infiltration and tissue reorganization^26^, leading to a chronic inflammatory condition, increased mucosal permeability and translocation of microbial products from the intestinal lumen to the bloodstream^27^. It is possible that neutrophils migrating to the site of HIV-1 infection release NETs after interaction with inflammatory mediators, translocated PAMPs, or even with HIV-1 particles or proteins. In fact, HIV-1 virions induce and are inactivated by NETs^8^, a finding that points to a possible role of these structures in the control of HIV-1 propagation. Also, NETs interact with macrophages and other immune cells in several tissues of SIV-infected macaques^28^, suggesting that NETs can also interact with HIV-1-infected cells in vivo.

Macrophages are critical cells for the establishment and maintenance of HIV-1 infection, due to their resistance to HIV-1-mediated cytopathic effects and ability to produce virus continuously, thus functioning as an HIV-1 reservoir^29^. Macrophages also contribute to virus propagation in lymphoid tissues through transmitting HIV-1 virions to CD4 + T cells^30^. Despite the potential NET interaction with infected cells in the sites of HIV-1 infection and replication, the impact of NETs on viral production in HIV-1-infected macrophages has not yet been studied.

It is reasonable to believe that NETs may interfere with the HIV-1 infection and production by macrophages. For example, NETs elicit macrophage release of inflammatory mediators able to regulate HIV-1 production^13,23^; NETs carry molecules that have been reported to modulate HIV-1 infection and replication, such as elastase^31^, cathepsin G^32^, myeloperoxidase^33^ and defensins^34^; the presence in the NETs of ligands of Toll-like receptors (e.g., calprotectins, histones, elastase), whose activation in HIV-1-infected cells reduce viral production^35–37^. Therefore, we evaluated here whether NETs can modulate HIV-1 replication in human primary macrophages and found that NETs released by neutrophils from either healthy donors or HIV-1-infected individuals restrict HIV-1 replication in human primary macrophages.

## Results

### NETs inhibit HIV-1 growth in macrophages

**Figure 1:**
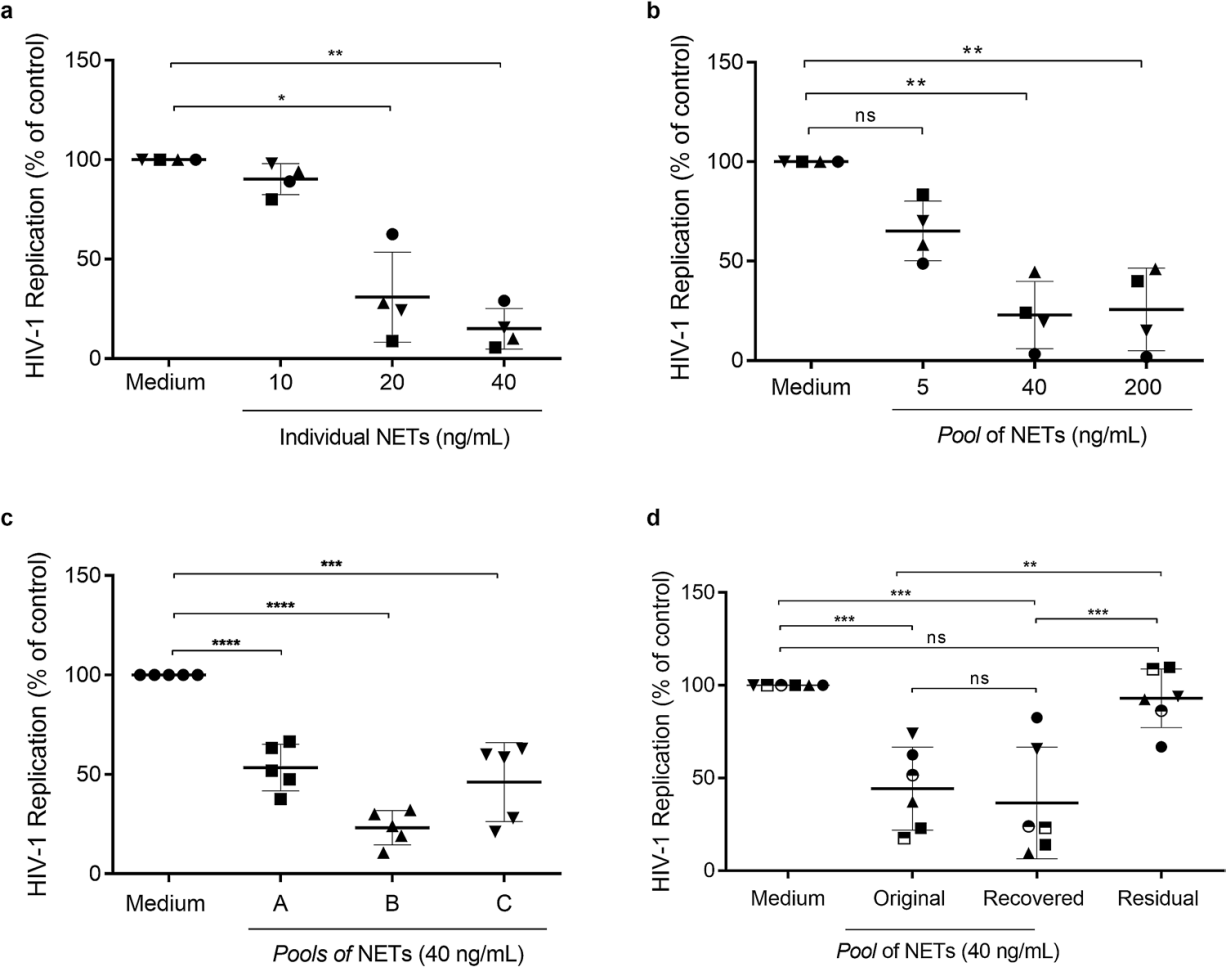
NETs inhibit HIV-1 production in macrophages. (**a**) Macrophages from healthy donors were infected in vitro and treated with NETs obtained from different individuals for 3 h, washed and maintained in culture (n = 4). (**b**) HIV-1-infected macrophages were treated with different concentrations of one pool of NETs during 3 h, washed and maintained in culture (n = 4). (**c**) HIV-1-infected macrophages were treated with three different pools of NETs during 3 h, washed and maintained in culture (n = 5). (**d**) One pool of five NETs was centrifuged and recovered using Centricon tubes of 100 kDa; thus, HIV-1-infected macrophages were treated with the original or with the recovered pool, or with the residual supernatant during 3 h; cells were washed and maintained in culture. In all these assays, HIV-1 replication was measured 12–14 days post-infection by quantifying the levels of HIV-1 p24 antigen in culture supernatants by ELISA. Data were analyzed with the Kruskal–Wallis test, with Dunn's post-test (**a**,**b**) and One Way ANOVA, with Turkey post-test (**c**,**d**). Each point represents a donor. Data represent means ± SD. Viral production in controls (cells incubated only with medium) in ng/mL: (**a**) = 13.8 ± 5.9; (**b**) = 17.5 ± 10.1; (**c**) = 14.7 ± 8.5; (**d**) = 13.06 ± 9.6. *p < 0.05; **p < 0.01; ***p < 0.001; ****p < 0.0001. *ns* non-significant.

Based on the findings that macrophages interact with NETs^13,22,23^, we investigated whether NETs would be able to modify the viral replication in HIV-1-infected macrophages. Initially, we obtained NETs from IL-8-activated neutrophils from healthy donors, which released a substantial amount of these structures (Neutrophils + IL-8: mean ± SD = 1145 ± 440 ng/mL of DNA; Neutrophils + medium: mean ± SD = 380 ± 356 ng/mL; n = 24; p < 0.0001). Next, to evaluate the effect of NETs on HIV-1 replication, HIV-1-infected macrophages from healthy donors were treated during three hours with varied concentrations of NETs obtained from different individuals, and we found that NETs reduced the viral production (Fig. 1a), reaching up to 80% of HIV-1 inhibition with 40 ng/mL of NETs .

To avoid potential variable results caused by possible constituent differences in NETs derived from individual donors, we analyzed HIV-1 replication in macrophages exposed to a pool of NETs, formed with NETs from at least five different donors, and observed that this first pool diminished the viral production at a similar degree to that promoted by individual NETs, with reductions close to 80% (Fig. 1b). We additionally assessed the anti-HIV-1 effect of three extra pools, randomly assembled with NETs from at least five different donors, and found a comparable viral inhibitory capacity between all them (Fig. 1c). Since the inhibitory effects on HIV-1 production were identical between pools or individual NETs, the next experiments were conducted using only pools of these structures.

During the activation processes, neutrophils may undergo degranulation and release several molecules into extracellular medium^38^. To rule out possible inhibitory effects from other neutrophil factors present in supernatant-containing NETs, but not related to these structures, pools of NETs were enriched using Centricon 100 centrifuge tubes, which allow the passage of molecules of molecular weight lower than 100 kDa. NETs retained on the membranes were recovered and resuspended in the original volume, and thus added to HIV-1-infected macrophages. We found that the recovered pool of NETs showed the same HIV-1 inhibition capacity as the original pool (Fig. 1d), whereas, in contrast, the residual supernatants, free of NETs, did not affect the virus production. These results suggest that the NET effects on HIV-1 replication in macrophages are independent of other unrelated neutrophil factors under 100 kDa possibly present in supernatant-containing NETs. Based on these findings, the next experiments were performed only with the original pools.

### NETs interact with macrophages without affecting cell viability

**Figure 2:**
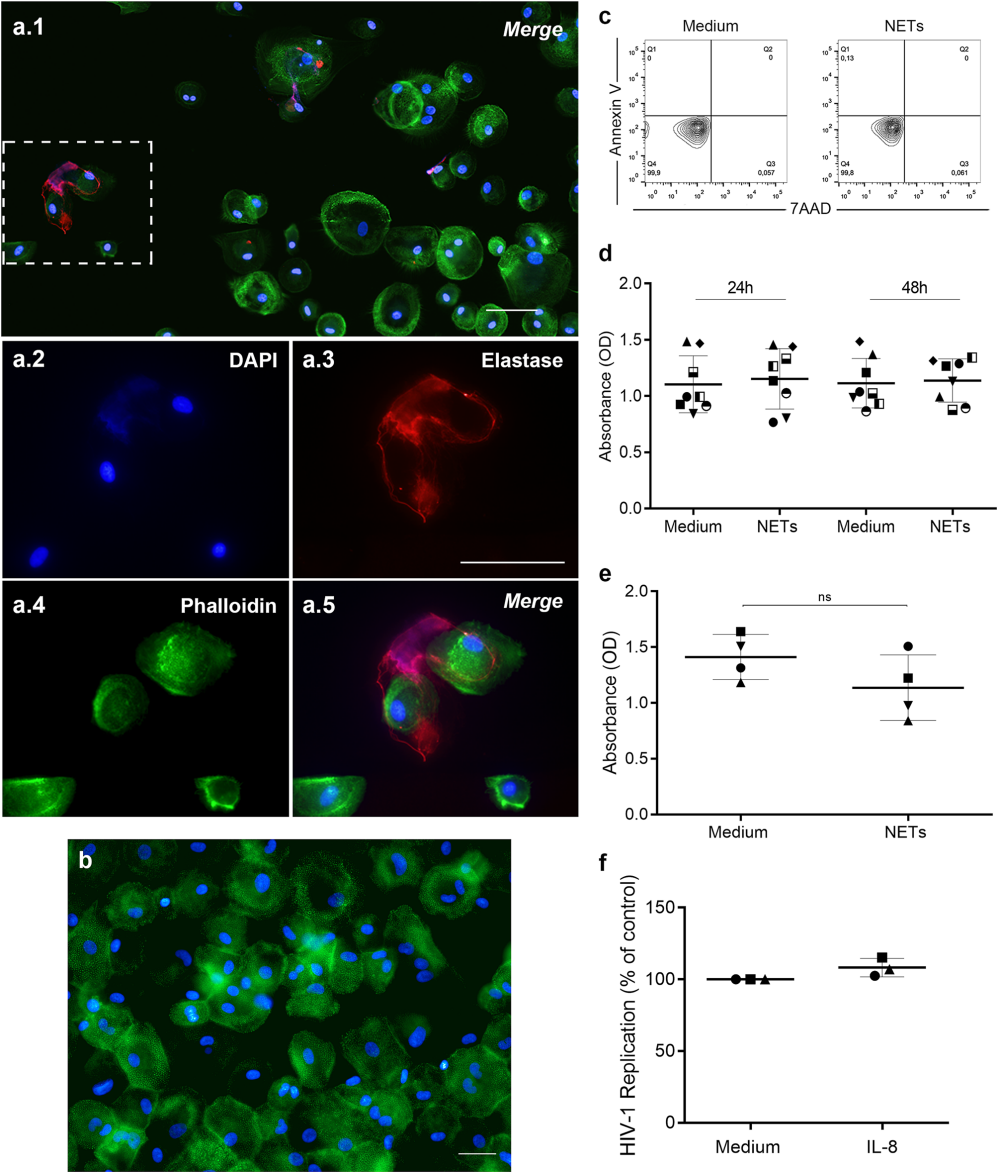
NETs interact with macrophages without affecting cell viability. (**a**) Interaction of NETs with macrophages from HIV-1 infected cultures. HIV-1-infected macrophages were exposed to NETs for 30 min and, after fixation, NETs were labeled with DAPI and anti-NE antibodies, while cells were visualized by phalloidin and DAPI labeling. Images (**a.2**) to (**a.5**) depict digital amplification of the area delimited by the dotted lines in (**a.1**), highlighting NET structures around the cells. Representative images of four different assays. Scale bar = 50 μm. (**b**) Removal of NETs by washing macrophages culture. Macrophages were incubated with NETs during 3 h, washed, fixed and labeled as described above. Observe that NET components were not detected. Representative image of three different assays, obtained after detailed and careful analysis of several microscopic fields by two independent investigators. Scale bar = 100 μm. (**c**) Viability of macrophages exposed to NETs. Macrophages were treated for 3 h with NETs, washed, and cell viability was evaluated 24 h later by flow cytometry (representative image of 2 independent experiments). Additionally (**d**), cell viability was assessed by XTT method after 24 h and 48 h or (**e**) in macrophages that had not been washed 14 days after NET treatment. *ns* non-significant. (**f**) HIV-1-infected macrophages were treated with IL-8 at a concentration equivalent to that carried over by the induced NETs, and no changes in HIV-1 production were observed in this condition.

At this point, some critical controls were performed. First, we noticed, by immunofluorescence, that the NETs colocalized with macrophages, suggesting an interaction with these cells (Fig. 2a). Second, we ascertained whether NETs were completely removed from macrophage cultures after washing (Fig. 2b), ruling out any possible NET direct inactivation effect on HIV-1 particles in our assays, as described elsewhere^8^. In addition, since it was recently described that NETs kill CD4^+^ and CD8^+^ T cells in SIV-infected nonhuman primates^28^, we evaluated the cell viability by two methods, and found that NET treatment did not affect macrophage survival, as assessed up to 14 days after exposure (Fig. 2c–e), indicating that reduction of viral production was not due to impairment of cell viability. Finally, to further ensure that HIV-1 inhibition promoted by NETs was not potentiated by the NET-inducing agent, HIV-1-infected macrophages were treated with IL-8 at a concentration equivalent to that carried over by the induced NETs, and we observed no changes in HIV-1 production in this condition (Fig. 2f)**.**

### Preservation of DNA–protein scaffold is essential for NET-mediated HIV-1 inhibition

**Figure 3:**
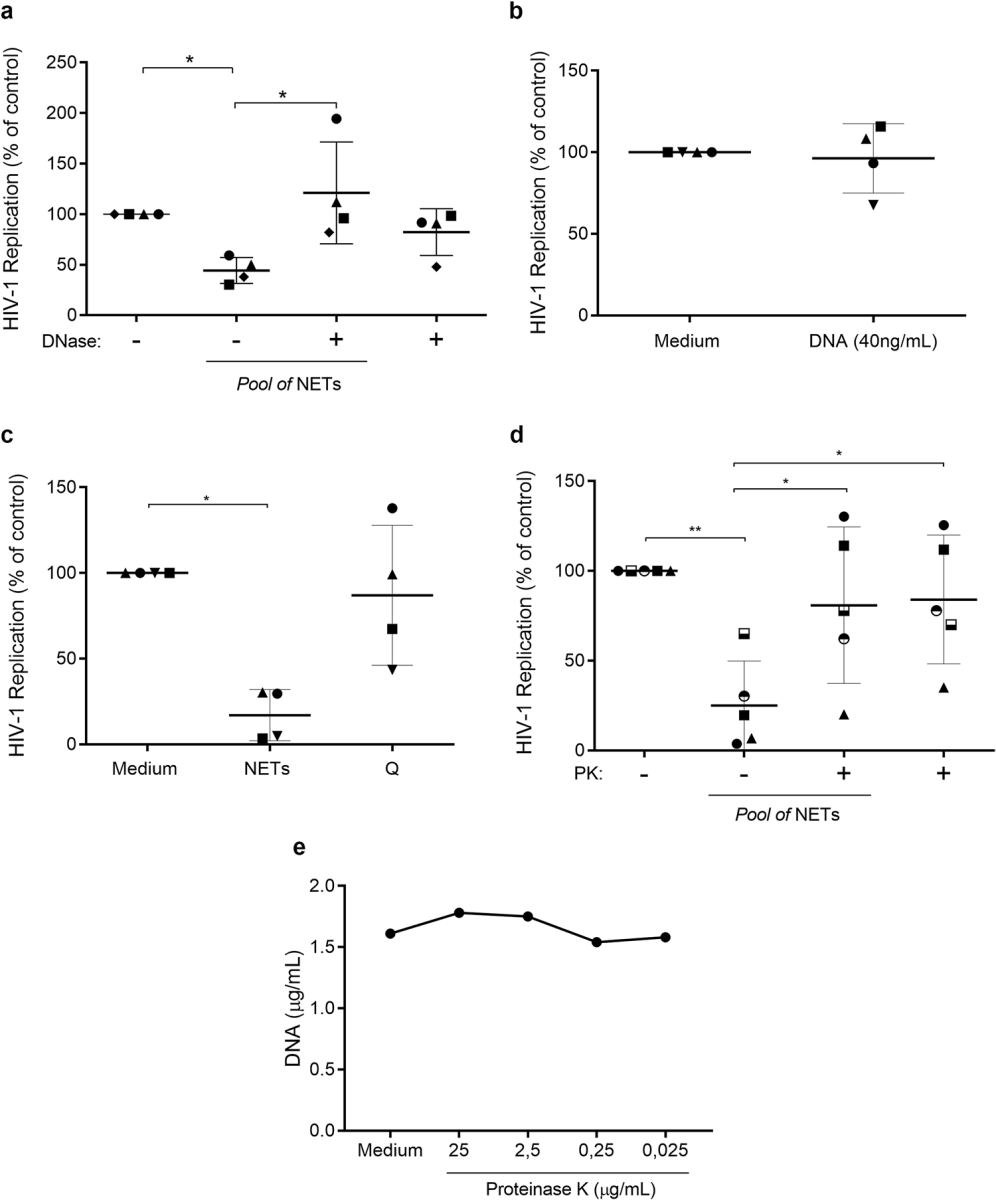
The integrity of DNA–protein scaffold is essential for viral inhibition mediated by NETs. HIV-infected macrophages were incubated with: (**a**) NETs treated or not with DNase (10 U/mL) or DNase alone; (**b**) salmon DNA (40 ng/mL); (**c**) NETs pre-incubated at 95 °C (Q) for 5 min; (**d**) NETs treated or not with Proteinase K (2.5 μg/mL) or Proteinase K alone for 30 min. In all these situations, cells were washed after 3 h treatments and kept in culture for 12–14 days, when HIV-1 replication was measured as described in the legend of Fig. 1. Data represent means ± SD [n = 4; (**a**–**c**) and n = 5 (**d**)]. Viral production in controls (cells incubated only with medium) in ng/mL: (**a**) = 16.5 ± 17.54; (**b**) = 14.4 ± 3; (**c**) = 15.8 ± 7.3; (**d**) = 29.3 ± 24.4. (**e**) NETs were treated with different concentrations of proteinase K for 30 min and DNA integrity measured with Picogreen (data are representative of three independent experiments). Each point represents a donor. Data were analyzed using Kruskal–Wallis test with Dunn post-test (**a**,**c**) and One Way ANOVA test, with Tukey's test (**e**). *p < 0.05; **p < 0.01.

Because NETs and some NET components possess microbicidal properties^13,39,40^, and based on our analysis showing a firm interaction between these structures and macrophages (Fig. 2a), we assessed how critical the maintenance of NET original chemical and physical structures would be for their action on HIV-1 replication. Thus, NETs were initially treated with DNase I and then added to HIV-1-infected macrophages, and we found that enzymatic DNA digestion significantly diminished the anti-HIV-1 effect of NETs (Fig. 3A). We further verified that purified salmon DNA did not modify the HIV-1 production (Fig. 3B), indicating that DNA is an essential NET component, but not sufficient, to promote HIV-1 inhibition in macrophages.

Heat treatment also abolished the NET anti-HIV-1 effects (Fig. 3c), suggesting that thermolabile NET-associated proteins could play a role in HIV-1 inhibition. We therefore investigated whether the anti-HIV-1 effect was dependent on the preserved structure of NET-associated proteins, and found that the ability of NETs to restrict HIV-1 replication was lost after enzymatic degradation of proteins (Fig. 3d). Before addressing this last hypothesis, proteolytic digestion of NETs was standardized in conjunction with DNA quantification, to assure that the nucleic acid integrity was not damaged during the proteolysis process. Treatment with proteinase K (2.5 μg/mL, 30 min) digested the NET-associated proteins without changing the DNA concentrations (Fig. 3e). These results indicate that the physical support provided by DNA and the structural maintenance of the DNA-associated proteins as well are critical for the NET inhibitory effect on HIV-1 replication. Studies are in progress in our laboratory to ascertain the contribution of NET-associated proteins to the anti-HIV-1 effects of these structures.

### NETs decrease HIV-1 DNA integration

**Figure 4:**
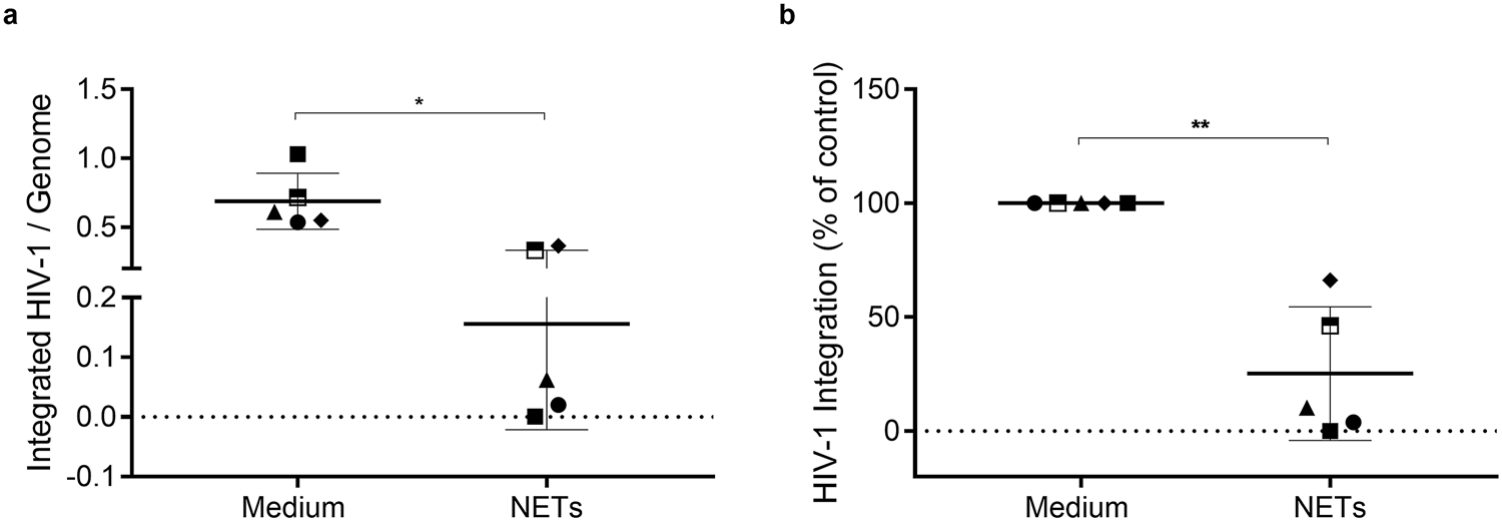
NETs decrease integrated HIV-1 provirus. HIV-1-infected macrophages were treated with NETs (40 ng/mL) for 3 h, and HIV-1 proviral integration was assessed by nested PCR 72 h later. (n = 5). (**a**) Number of integrated provirus per genome; data represent means ± SD of each treatment. (**b**) Normalized data of (**a**). Data were analyzed using Paired t-test. *p < 0.05; **p < 0.01.

Because NETs inhibit HIV-1 production when added to HIV-1-infected macrophages just after the cellular infection, we asked whether NETs would be interfering in a post-entry event. We thus investigated the ability of NETs to modulate the integration of the HIV-1 proviral DNA to macrophage genome, an early step of the HIV-1 replicative cycle. Therefore, HIV-1 integration was analyzed by nested PCR at 72 h after NET addition, as described by Liszewski et al.^41^, and we found a significant decrease in the levels of integrated HIV-1 provirus in NET-treated HIV-1-infected cells (Fig. 4). This result suggests that the NET-inhibition of HIV-1 production in macrophages can be explained, at least in part, by their ability to reduce the integration of HIV-1 DNA into the cell genome.

### NETs reduce HIV-1 replication fitness

**Figure 5:**
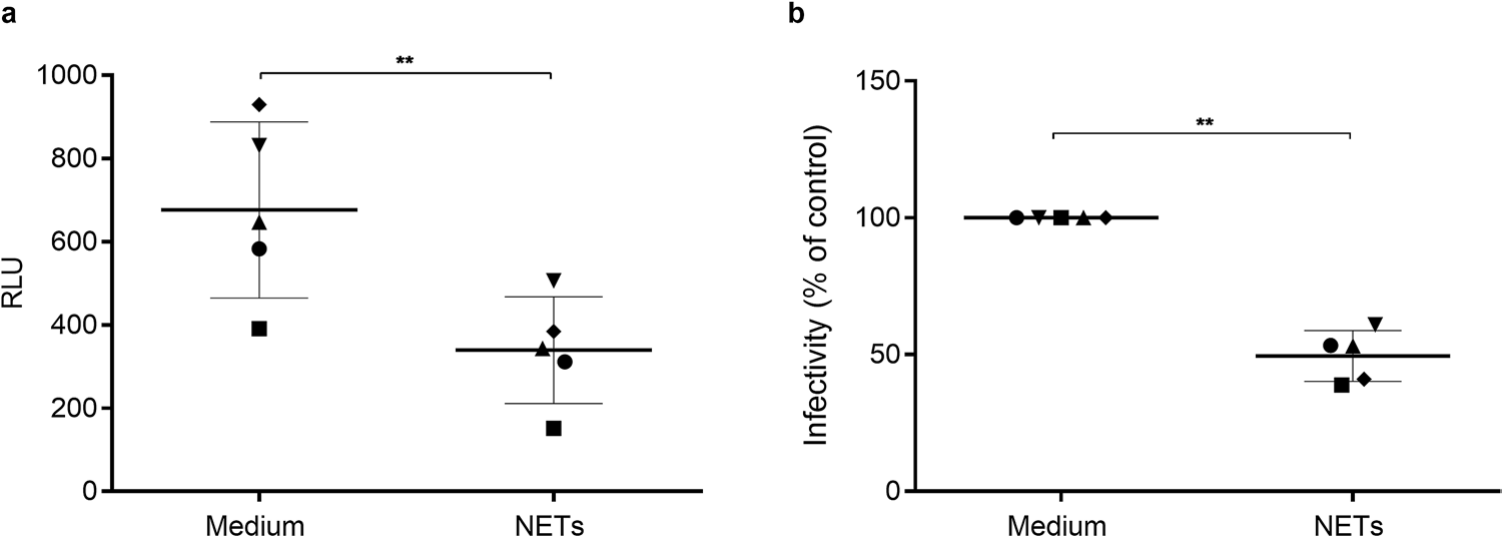
NETs reduce HIV-1 replication fitness. TZM-bl cells were exposed to standard concentrations of viral particles obtained after 12 days culture of HIV-1-infected macrophages exposed or not to NETs (40 ng/ml). After 48 h, TZM-bl cells were lysed and luciferase activity measured. (**a**) Relative luminescence units (RLU); data represent means ± SD. (n = 5). (**b**) Normalized data of (**a**). Each point represents a donor. Data were analyzed using Student’s t test. **p < 0.01.

We next asked whether the residual viruses released by macrophages exposed to NETs retained their infective capacity unchanged. To address this issue, standardized amounts of viral particles were added to TZM-bl cells for evaluation of viral infectivity. We observed that virions that emerged from macrophages exposed to NETs showed significant loss of their infective capacity (Fig. 5). Based on this finding, we believe that NET-mediated inhibition of HIV-1 infection relies not only on the reduction of virus production, but also on the concomitant loss of virus fitness, which can lead, eventually, to the weakening of virus propagation.

### NETs increase β-chemokine production by macrophages

**Figure 6:**
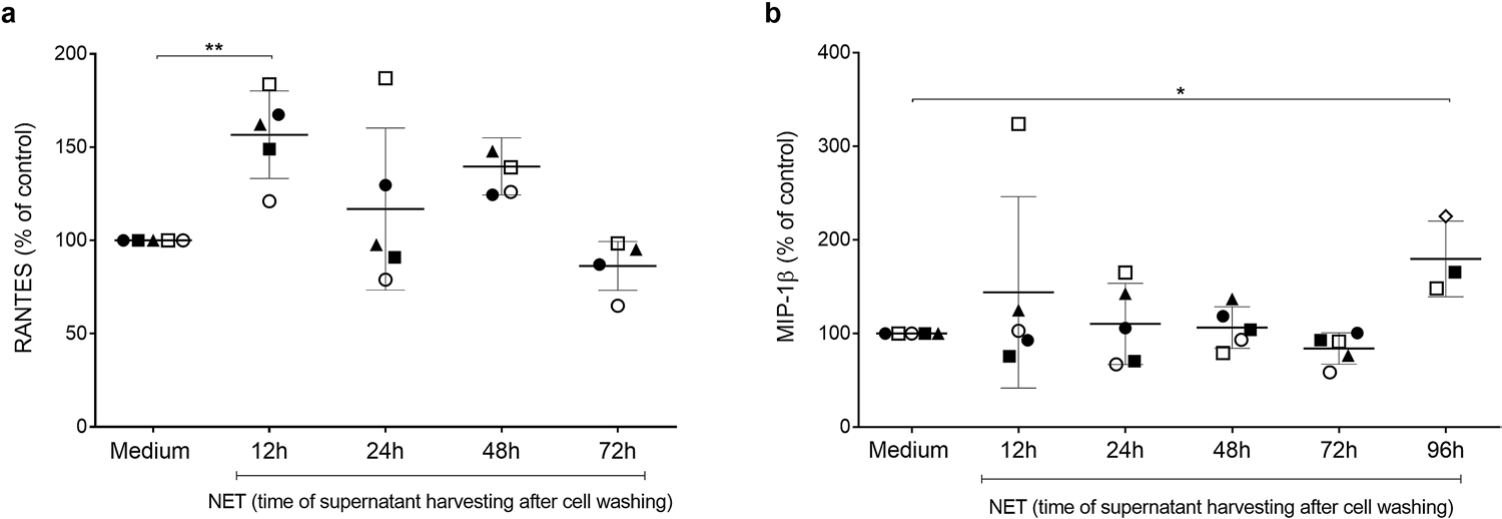
NETs elicit macrophage production of Rantes and MIP-1β. HIV-1-infected macrophages were left untreated (medium) or treated with NETs (40 ng/mL) for 3 h, washed and maintained in culture during the indicated times. Supernatants were then collected, and the concentrations of Rantes (**a**) and MIP-1 β (**b**) were measured by ELISA. Each point represents a donor. Data represent means ± SD, which were analyzed using Kruskal–Wallis test with Dunn post-test. *p < 0.05; **p < 0.01.

We evaluated whether NETs would induce macrophage secretion of β-chemokines, taking into consideration that these mediators are potent inhibitors of HIV-1 infection^36^. Indeed, NETs enhanced macrophage release of Rantes and MIP-1β, whose production peaked 12 h and 96 h after NETs addition to cells, respectively (Fig. 6). These data, although showing some variability and moderate effects, suggest that both molecules contribute to NET-elicited restriction of HIV-1 production in macrophages.

### Neutrophils from HIV-1-infected patients release NETs, which are present in the patients’ plasma

**Figure 7:**
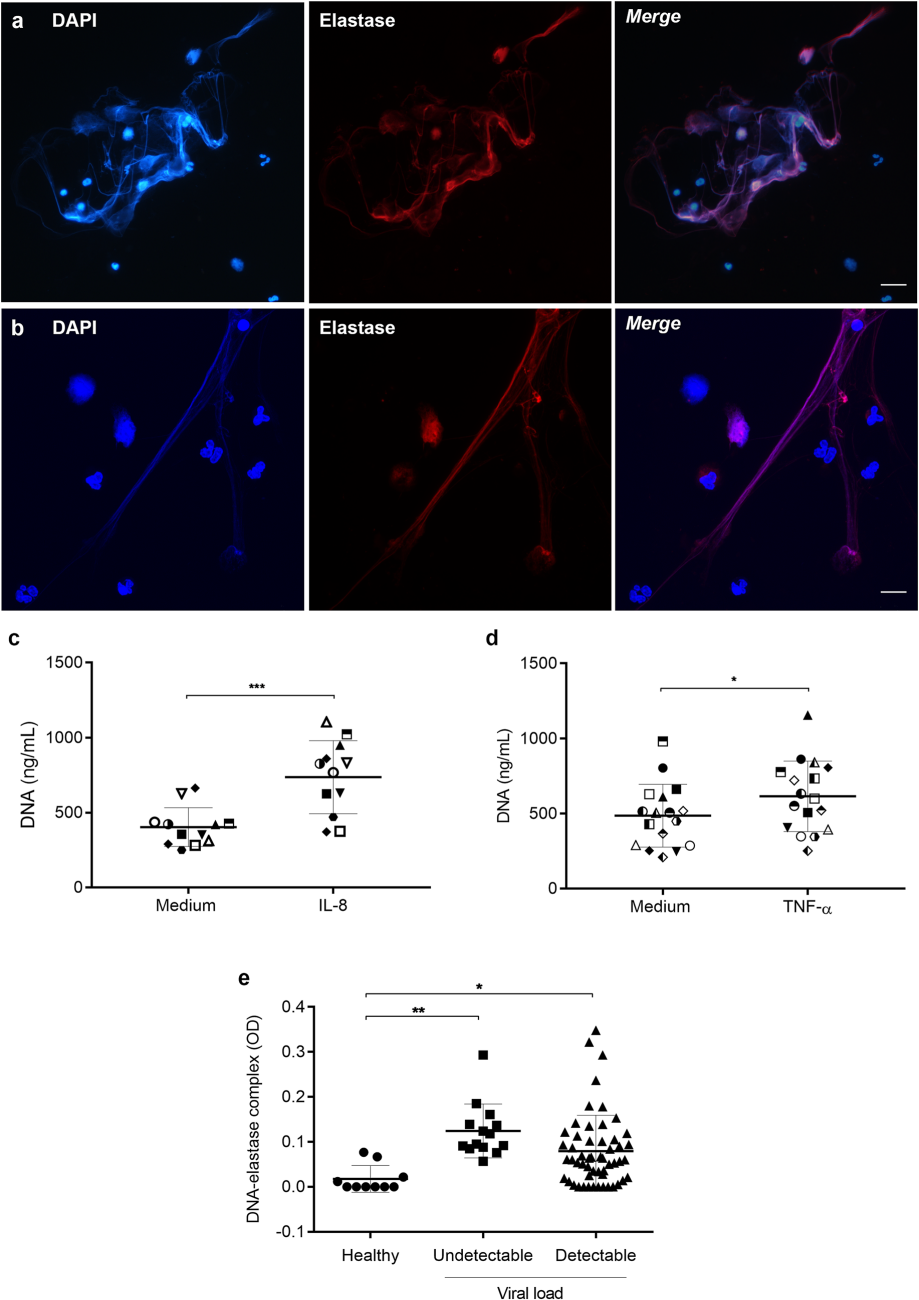
Neutrophils from HIV-1 patients release NETs, which are present in patients’ plasma. (**a**) Neutrophils from HIV-1 patients or (**b**) from healthy donors were activated with IL-8 (50 ng/mL) for 3 h at 37 °C, fixed and labeled with DAPI and anti-NE antibodies. Merged images show the colocalization of the two markers and the network morphology of NETs. Images are representative of at least 6 (HIV-1 patients) or 4 (healthy donors) assays. Scale bar, 10 µm. (**c**,**d**) Neutrophil from HIV-1 patients were stimulated with IL-8 (50 ng/mL; n = 12) or TNF-α (20 ng/mL; n = 17) and NET release was quantified with the Picogreen dsDNA kit. Each point represents a donor. Data were analyzed using paired Student’s t test. (**e**) Quantification of DNA-elastase complexes in the plasma of HIV-1-infected patients. Data were analyzed using ANOVA. Viral loads in HIV-1 RNA copies/mL: Undetectable: < 40; Detectable: > 40. *p < 0.05; **p < 0.01; ***p < 0.001.

HIV-1 infection leads to disruption of the intestinal mucosal barrier, with the consequent translocation of bacterial products to the circulation, local inflammation, and chronic activation of the immune system^27^. Considering that bacterial products and inflammatory mediators can activate neutrophils and induce NET release, we investigated whether HIV-1-infected patients present elevated plasma levels of NETs, and whether neutrophils from HIV-1-infected patients release NETs upon activation in vitro with pro-inflammatory mediators. In fact, we found that neutrophils from HIV-1-infected patients do release NETs, as can be observed through immunofluorescence staining showing extracellular DNA fibers colocalizing with NE (Fig. 7a), in a similar way to healthy subjects (Fig. 7b), and by the significant amount of NET DNA released upon neutrophil activation with IL-8 (Fig. 7c) or TNF-α (Fig. 7d). Also, pronounced amounts of DNA-NE complexes were found in patients’ plasma samples, relative to plasma from healthy individuals, regardless whether their HIV-1 viral loads were detectable or not (Fig. 7e). Altogether, these findings suggest that neutrophils from HIV-1-infected patients are prone to release NETs, which can be found in their blood. Table 1 shows the viral load, CD4^+^ and CD8^+^ T cell counts and treatment status of the HIV-1-infected patients at the time of blood sampling.

**Table 1:** Laboratory features of HIV-1-infected individuals.

HIV-1 viral load	N	Viral load (mean ± SD)	CD4^+^ T cell count (mean ± SD)	CD8^+^ T cell count (mean ± SD)	Sex (F:M)
Undetectable	14	–	592 ± 206	823 ± 227	9:5
Low	14	831 ± 917	505 ± 347	1.312 ± 680	5:9
Medium	21	36.646 ± 20.679	220 ± 155	904 ± 372	13:8
High	18	453.459 ± 975.020	77 ± 60	768 ± 446	7:11

Healthy donors were selected to match the age and sex distribution of the patients.

All the patients were under combined antiretroviral treatment.

*F* female, *M* male.

### NETs released by neutrophils of HIV-1-infected patients reduce HIV-1 replication in macrophages

**Figure 8:**
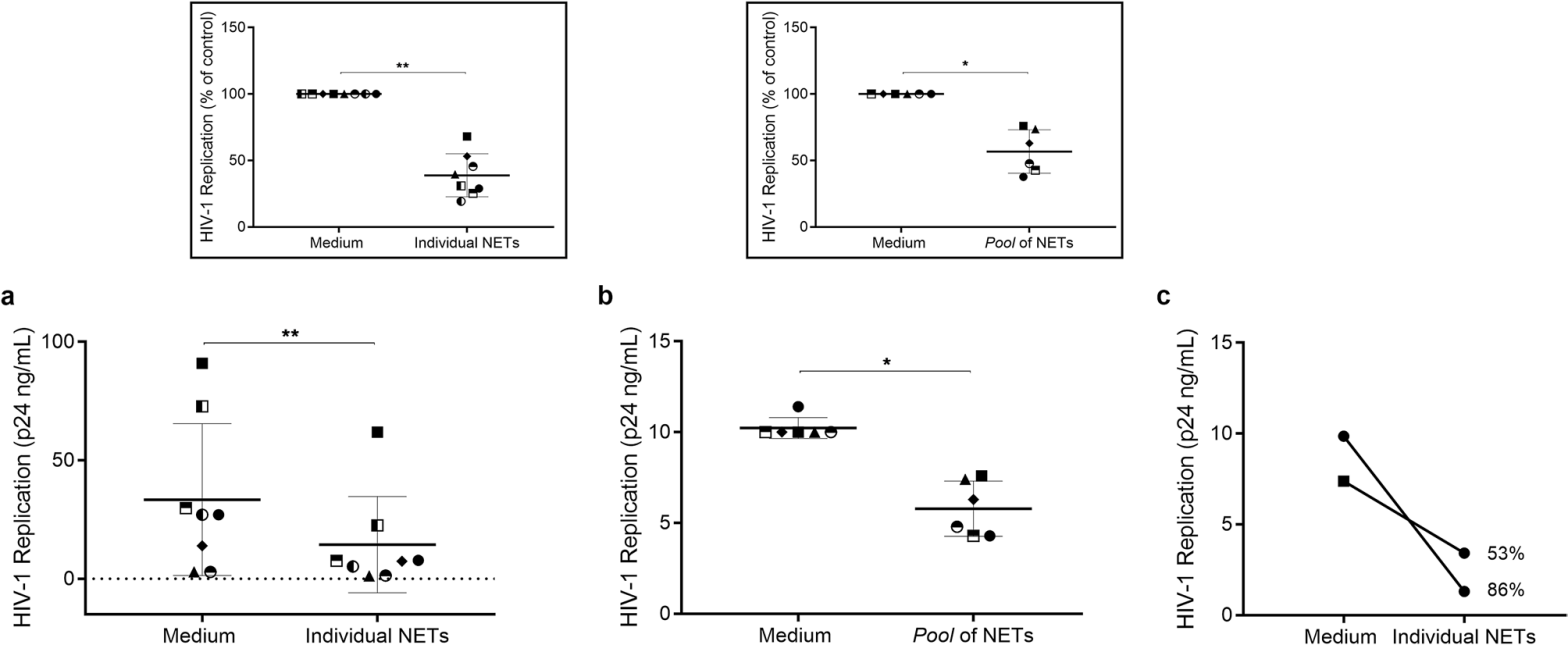
NETs released by neutrophils from HIV-1-infected patients inhibit HIV-1 replication in macrophages. Macrophages from healthy donors were infected in vitro and treated for 3 h with (**a**) individual NETs obtained from HIV-1 patients, or (**b**) a pool of these NETs, washed and maintained in culture during 12–14 days, when HIV-1 replication was measured as described in the legend of Fig. 1. Inserts represent normalized data from (**a**) and (**b**). (**c**) Macrophages from two HIV-1-infected patients were infected in vitro and exposed to NETs obtained from their own neutrophils; the following procedures were the as described above. Numbers mean percentage of inhibition relative to respective control. (**a**,**b**) Data were analyzed using paired Student’s t test. *p < 0.05; **p < 0.01.

Based on our findings that NETs released by neutrophils from healthy individuals inhibit HIV-1 replication (Fig. 1), and that the NETs have been detected in intimate contact with HIV-1 target cells^28^, we analyzed whether NETs from neutrophils of HIV-1-infected patients would also be able to restrict HIV-1 production. Using the same protocol as described for Fig. 1, we found that these NETs are indeed endowed with the ability to restrain HIV-1 replication in macrophages (Fig. 8). Of note, NETs from two infected individuals reduced HIV-1 replication in their own macrophages (Fig. 8c). These results obtained with clinical samples suggest that NETs may contribute to diminish viral production by HIV-1-infected macrophages, thus preventing HIV-1 dissemination to other HIV-1 target cells.

## Discussion

The involvement of NETs in different inflammatory or infectious scenarios has widely been documented, conveying detrimental or beneficial effects to the host. In fact, it has been suggested that aberrant production of NETs might be implicated in the development of pathological outcomes, such as autoimmune diseases, cancer, thrombosis and tissue damage^42^. On the other hand, NETs have been shown to control the growth and propagation of a variety of pathogens, such as bacteria^1,23,39,43,44^, parasites^7,45,46^, fungi^2,47–49^, and viruses^8,28,50–55^.

Regarding the possible impact of NETs on HIV-1 infection, the only two published studies up to now, to our knowledge, limited their observations to the NETs effects on the viability of the HIV-1 particles. Thus, Saitoh et al.^8^ reported that HIV-1 virions induce NET release from neutrophils of healthy individuals and that these structures trap and render the virus non-infective. More recently, Barr et al.^56^ described that neutrophils from female genital tract release NETs that capture and inactivate HIV-1 particles, thus preventing new infections of CD4^+^ T cells.

Here, we explored the effect of NETs on viral replication upon treatment of HIV-1-infected macrophages with these structures. We found that individual NETs or NETs combined in pools, either from healthy donors or from HIV-1-infected patients, inhibited viral growth. Importantly, the maintenance of the NET DNA-proteins structural composition was essential in this phenomenon. We believe that the NET-induced reduction of HIV-1 provirus integration and the loss of viral infectivity explain the diminished virus production by macrophages, with the contribution of β-chemokines. Given the different experimental approaches between our work and the two studies mentioned above (direct exposure of virion particles to NETs^8,56^), we believe that our study brings novel and significant findings upon describing the impact of NETs on the control of HIV-1 replication in one of its target cells.

Macrophages can acquire microbicidal activity against intracellular pathogens following uptake of neutrophil granules^19,57^, or after interaction with molecules present in these compartments^17,21,58^. Therefore, once detecting HIV-1 inhibition by NET-containing supernatants, we asked whether other products spontaneously secreted by neutrophils in the culture supernatants, but not associated with the released NETs, would have contributed to the observed anti-HIV-1 effect. Thus, infected macrophages were exposed to NETs previously recovered on 100 kDa filters, and we found that the concentrated NETs (that might contain other molecules larger than 100 kDa) preserved their ability to inhibit HIV-1 replication, while the NET-free fraction had no effect on viral growth. We then assumed that, in our system, the NET modulating effect on HIV-1 production in macrophages depends only on NET interaction with infected macrophages, without any apparent contribution of neutrophil soluble molecules.

Consistent with findings reported by other groups showing that human primary macrophages and THP-1-derived macrophages treated with NETs produced the β-chemokines MIP-1α, MIP-1β and Rantes^13,59^, we observed that primary macrophages exposed to NETs released increased amounts of Rantes and MIP-1β. Taking into account that we and other authors have shown the potent anti-HIV-1 effect of β-chemokines^35,36,60^, this result suggests that NETs not only can activate the macrophages, but also that these anti-HIV-1 agents probably contribute to the restricted HIV-1 production in macrophages exposed to these structures (of note, NETs did not enhance macrophage release of IL-10, a cytokine also able to inhibit HIV-1 replication in macrophages^35,60^; data not shown). How NETs elicit macrophage production of β-chemokines is not clear, but it might be due to the cellular response to one or more NET constituents, since other authors showed, for example, that α-defensin induces MIP-1α and MIP-1β synthesis in human macrophages^61^.

We found that the maintenance of original chemical and physical structures of NETs is essential for their capacity to mediate HIV-1 inhibition, as heat, DNAse or proteinase K treatments abolished this effect. Similar observations have been made by other authors who have highlighted that the NET’s DNA is an important scaffold for active proteins, concentrating these molecules in a punctual manner instead of being freely diffused in the aqueous environment^40,62^. Regarding the effect of NET-associated proteins, investigations are currently in progress in our laboratory to determine their possible contribution for the NET-mediated inhibition of HIV-1 infection, as described here.

HIV-1 replication shows different dynamics in macrophages compared to CD4^+^ T lymphocytes, in part due to the low levels of deoxyribonucleotide triphosphate (dNTPs) pools caused by the activity of the restriction factor Sterile alpha motif and histidine/aspartic acid domain protein 1 (SAMHD1^63^). In accordance with Bejarano et al.^64^, the HIV-1 entry in macrophages occurs around 8 h post infection, while the reverse transcription and nuclear import of the HIV-1 genome are completed between 60 and 72 h post-infection. Since NETs are added to cells after HIV-1 entry, the marked decline in viral production observed 12–14 days post treatment is, naturally, a consequence of a disruption in a different step of the cycle. The viral integration into the cell genome is a crucial early step in the HIV-1 replicative cycle and is required for productive infection. Considering that *Alu* is a repeated element in the human genome that favors HIV-1 DNA integration^65,66^, we adopted the method described by Liszewski et al.^41^ to evaluate this phenomenon in NET-treated HIV-1-infected cells. The reduced levels of HIV-1 integration observed at 72 h post infection show that the infection process was affected in an early post-entry stage of the viral cycle, explaining, at least partially, the diminished HIV-1 production observed in our model. Additional studies should be done to elucidate the mechanisms by which NETs reduce the levels of HIV-1 integration, such as, for example, whether NETs act during the reverse transcription and/or nuclear import of the viral genome, or even directly on the proviral integration process.

During HIV-1 infection, virtually all cells of the immune system undergo functional changes^67,68^. In fact, neutrophils may exhibit abnormalities, such as impaired chemotaxis, phagocytosis and oxidative capacity, and negative regulation of IL-8 receptors on plasma membrane^69^. Given these potential defects, we then considered as relevant to evaluate the NET production in this infection scenario and collected a group of data indicating that neutrophils from HIV-1-infected patients release NETs. Furthermore, we detected increased levels of DNA-NE complexes in plasma samples from HIV-1-infected patients, similarly to what has been observed in pathological conditions^62^ and in several infectious diseases^9,10,12,70–72^. Likewise NETs released by neutrophils of healthy donors, NETs from neutrophils of HIV-1-infected patients also restrain HIV-1 production in macrophages. Overall, our data suggest that NET formation can occur in HIV-1-infected patients, and, once released in tissues containing infected macrophages, can potentially restrict HIV-1 production and then viral dissemination for other target cells. In this sense, Sivanandham et al.^28^ reported that isolated neutrophils of SIV-infected nonhuman primates released NETs, which were observed interacting with immune cells, including macrophages, in several tissues of these animals.

An open question remains, to be solved with more studies, whether NET formation is beneficial to the HIV-1 host, as can be inferred from studies showing that NETs trap and destroy HIV-1 particles^8,56^ and inhibit HIV-1 replication (our present findings), or detrimental, as can be reasoned by investigations reporting that NETs capture and kill immune cells^28^. We suppose that both situations can occur, depending on a balance between a moderate or an excessive NET formation in tissues in which HIV-1-infected cells are actively producing viral particles. The plasma and local concentration of NETs may vary according to the number of neutrophils infiltrating the inflamed site (neutrophil swarming^73^) and the neutrophil population releasing NETs. In other words, regarding HIV-1 infection, the NET concentration will depend on the magnitude of neutrophil swarming in tissues where HIV-1-infected cells are producing and releasing viruses.

Finally, our findings indicate that more studies should be performed to explore whether NETs contribute to control infection and viral replication in other HIV-1-target cells, such as dendritic and CD4^+^ T cells, and also for characterizing further mechanisms underlying the ability of these structures to restrain HIV-1 production in infected cells.

## Methods

### HIV-1 isolates, reagents and ELISA kits

HIV-1 infections were performed using the CCR5-dependent isolate HIV-1_Ba-L_ (donated by the AIDS Research and Reference Reagent Program, NIH, Bethesda, MD), as we described elsewhere^74^. Recombinant IL-8 was purchased from BioLegend, and the anti-neutrophil elastase (NE) antibodies were acquired from Calbiochem. The DNase I and salmon sperm DNA were obtained from Promega, the proteinase K was purchased from Invitrogen, and the anti-DNA peroxidase-conjugated antibody from Immunotools. The HIV-1 p24 ELISA kits were acquired from Sino Biological, and Rantes, MIP-1α and MIP-1β ELISA kits from R&D Systems.

### Isolation and culture of primary human neutrophils, macrophages, and cell lines

Human neutrophils were obtained from buffy coats of healthy adult blood donors by density gradient centrifugation (Ficoll-Paque Premium, GE Healthcare Life Sciences). Neutrophils were further separated from erythrocytes by spontaneous sedimentation through 3% dextran 500 (Sigma), followed by hypotonic lysis of remaining red cells. Purified neutrophils (> 95% as determined by flow cytometry analysis using anti-CD16; data not shown) were resuspended in RPMI 1640 medium (Sigma) and kept in ice until use. Human monocyte–derived macrophages were prepared from peripheral mononuclear cells (PBMCs) through adherence onto plastic plates, as we described elsewhere^35^. Briefly, after density gradient centrifugation from buffy coats, PBMCs were plated onto 96-well plates (Costar) in Dulbecco’s modified Eagle’s medium (DMEM; LGC Bio) containing 10% normal human serum (Sigma) and penicillin–streptomycin (Gibco) during 7–10 days for monocyte differentiation into macrophages. Next, non-adherent cells were removed, and the macrophages were maintained in standard conditions in DMEM supplemented with 5% human serum. Macrophage purity was > 90%, as determined by flow cytometry analysis using anti-CD3 and anti-CD68 monoclonal antibodies (BD Bioscience). TZM-bl cells (obtained through the AIDS Research and Reference Reagent Program, Dr. John C. Kappes, Dr. Xiaoyun Wu and Tranzyme Inc) were maintained with DMEM low-glucose with 10% heat-inactivated fetal calf serum and antibiotics. Neutrophils, macrophages and plasma from HIV-1-infected patients were obtained using the same protocols as above, after collecting 8–10 mL of peripheral blood by venipuncture.

### NET induction

Neutrophils (2 × 10^6^/well/400 μL) from healthy blood donors or from HIV-1-infected patients were stimulated with IL-8 (50 ng/mL) or with TNF-α (20 ng/mL) for 3 h at 37 °C in RPMI 1640 medium, without serum. Then, supernatants containing NETs were removed through extensive pipetting, centrifuged at 400 × *g* for five min to remove residual neutrophils, and NET DNA was quantified by using Quant-iT PicoGreen dsDNA Assay (Invitrogen). The retrieved NETs were preserved at − 80 °C until further use.

### Immunofluorescence assays

Neutrophils (5 × 10^5^/well in Permanox chamber slides (Nalge Nunc) were stimulated with IL-8 and immediately fixed with 4% formaldehyde (FA, Merck) for 30 min and rinsed three times with PBS. Next, samples were blocked with human serum for 1 h and incubated with anti-elastase monoclonal antibodies (1:250; Calbiochem) for two hours. Samples were washed and incubated with secondary antibodies coupled to Alexa546 (Life Technologies) for 30 min, rinsed twice with PBS and mounted with ProLong Diamond anti-fade containing DAPI (Life Technologies). For NET-macrophage interaction assays, these cells were exposed to NETs (500 ng/mL) for 30 min, fixed with FA (4%, 30 min), washed and blocked as described above. Samples were then stained with anti-elastase and Alexa 546-labeled secondary antibodies and Phalloidin-488 (Invitrogen) for 30 min, and then mounted on medium containing DAPI, as above. All staining processes were performed at room temperature (RT). Slides were examined using confocal (Leica DMi8) and fluorescence (Zeiss Ax10) microscopes, and LasX and AxioVision softwares, respectively. Brightness, contrast and color of digital images were adjusted with Adobe Photoshop CS5 v12.0 program (Adobe Systems Inc.).

### HIV-1 infection and effects of NETs on HIV-1 replication

Macrophages were exposed overnight to viral suspensions containing 5–10 ng/mL of HIV-1 p24 Ag, as we described previously^35^. Cell monolayers were washed, replenished with fresh medium and maintained under standard culture conditions. Viral replication was measured 12–14 days post-infection by quantifying the levels of HIV-1 p24 antigen in culture supernatants using ELISA kits (Sino Biological). To evaluate the effects on HIV-1 production, individual NETs or pool of NETs (5–6 individual NETs/pool) were added to HIV-1-infected macrophages immediately after infection for 3 h in the absence of serum, and then cells were washed to remove residual NETs and kept in culture for 12–14 days, when viral replication was quantified. In another set of experiments, HIV-1-infected macrophages were treated with NETs recovered after centrifugation in Centricon 100 tubes (Millipore), and viral replication was evaluated as already described. As a control, equivalent amounts of IL-8 possibly carried over by NETs were added to infected macrophages.

### Cell viability assays

The viability of macrophages treated with NETs was determined using the 2,3-bis (2-methoxy-4-nitro-5-sulfophenyl)-2H-tetrazolium-5-carboxanilide (XTT, Sigma) method, as described elsewhere^75^, and also through Annexin-V binding and Propidium Iodide (PI) staining, followed by flow cytometry using a BD Canto II cytometer equipped with BD software FACSDiva (BD Bioscience, USA). The data obtained were analyzed using FlowJo v10 software, Tree Star Inc.

### Degradation of NET components and HIV-1 replication assays

For DNA digestion, NETs were incubated with DNase I (10 U/mL) for 30 min at 37 °C. Then, enzymatic digestion was stopped by adding EDTA (2.4 mM), and DNA degradation was confirmed by Picogreen method. Protein degradation was performed by treating NETs with proteinase K for 30 min, and protein digestion was verified by electrophoresis on polyacrylamide gels (SDS/PAGE) and then revealed by silver staining. To assess DNA preservation after NET treatment with proteinase K, samples were also analyzed using Picogreen method. Then, DNase- or proteinase K-treated NETs were added to HIV-1-infected macrophages, and viral replication was evaluated as above. As an additional control, HIV-1 production was also evaluated in HIV-1 infected macrophages exposed to salmon sperm DNA (40 ng/ml).

### Effects of NETs on HIV-1 DNA integration

To assess this effect, HIV-1-infected macrophages were treated or not with NETs for three hours, washed and maintained in culture for more 72 h. Next, total DNA was isolated using AllPrep DNA/RNA Mini kit (Qiagen) and quantified by Qubit, using Qubit dsDNA HS Assay Kit. First, we generated a standard curve with J-lat cells, which are Jurkat-based cell line containing a full-length integrated HIV-1 genome^76^, for beta-globin and integrated HIV-1. Next, to evaluate HIV-1 integration per cell, we determined the number of human genomes in our samples by real-time quantitative PCR (qPCR) for beta-globin and performed a nested-PCR, as described by Liszewski et al.^41^. The qPCR for beta-globin was performed using PrimeTime Gene Expression Master Mix 2X (Integrated DNA Technologies) with the specific pair of primers: β-Globin (Forward) 5′-CCC TTG GAC CCA GAG GTT CT-3′; β-Globin (Reverse) 5′-CGA GCA CTT TCT TGC CAT GA-3′ and β-Globin Probe 5′-GCG AGC ATC TGT CCA CTC CTG ATG CTG TTA TGG GCG CTC GC-3′. The qPCR was performed in an Applied Biosystems 7500 Real-Time PCR System (Thermo Fisher) using the following cycling protocol: 95 °C for three min, then 40 cycles of 95 °C for 15 s and 60 °C for one min. The first round of PCR for HIV-1 integration was performed using one primer that anneals to *Alu* (Forward: 5′ GCC TCC CAA AGT GCT GGG ATT ACA G-3′) and other that anneals to HIV *gag* (nucleotides 1505–1486; Reverse: 5′ GTT CCT GCT ATG TCA CTT CC-3′). The PCR conditions were: 94 °C for two min, then 40 cycles of 94 °C for 30 s, 50 °C for 30 s and 72 °C for 3 min 30 s. The second round of PCR quantifies HIV-1-specific products by using primers that anneals to the regions R and U5 in the HIV long terminal repeat. In order to do so, a qPCR was performed as described above, using the specific primers: RU5 (R Forward) nt 518–539: 5′-TTA AGC CTC AAT AAA GCT TGC C-3′; RU5 (U5 Reverse) nt 647–628: 5′-GTT CGG GCG CCA CTG CTA GA-3′; RU5 wild type Probe nt 584–559: 5′-CCA GAG TCA CAC AAC AGA CGG GCA CA-3. Then, the HIV-1 integration was calculated based on the standard curve established with J-lat cells considering DNA mass.

### Infectivity assays

To address whether NETs interfere with HIV-1 replication fitness, HIV-1-infected macrophages were treated with NETs for three hours in 6-well culture plates, washed and, after 12 days, culture supernatants were collected, centrifuged at 3000 × *g* and filtered on membranes with 0.45 μm pores. The HIV-1-containing supernatants were then centrifuged on Centricon filter devices with YM-100 membranes (Millipore) to concentrate and quantify viral particles by p24 ELISA. Then, this viral suspension (10 ng/mL p24 Ag) was added to TZM-bl cells (10^5^ cells/well/96-well plate) in the presence of DEAE-Dextran (15 µg/mL; Sigma). Viral multiplication was analyzed 48 h later, through measuring luciferase activity with the Bright-Glo reagent (Promega), following the manufacturer's instructions.

### Production of β-chemokines by NET-treated macrophages

Macrophages were treated with NETs and kept in culture under standard conditions, and cell culture supernatants were harvested at different time-points to measure the production of Rantes, MIP-1α and MIP-1β by ELISA (R&D Systems).

### Plasma and macrophages from HIV-1-infected patients

Plasma and blood samples from HIV-1-infected patients were obtained at the Nova Iguaçu General Hospital (RJ, Brazil), which is a referral center from the Ministry of Health for the treatment, care and follow up of people living with HIV. Plasma samples were stored at − 80 °C immediately after collection, and macrophages were obtained from patients’ PBMCs by density gradient, as described above. Table 1 shows the viral load, CD4^+^ and CD8^+^ T cell counts and treatment status of the HIV-1-infected patients at the time of blood sampling.

### Quantification of plasma DNA-elastase complexes

These complexes were quantified using capture ELISA. Briefly, 96-well microtiter plates were coated with 2.5 µg/mL of anti-NE antibodies (Calbiochem, Cat-No.481001) overnight at 4 °C. Wells were washed with PBS, blocked in BSA 2% for 2 h at RT and washed again. Then, plasma samples diluted 1:2 in sterile PBS were added to the wells and incubated overnight at 4 °C. Next, wells were washed with Tween 20 (0.05%) in PBS and 1.5 µg/mL of anti-DNA-peroxidase conjugated antibody (Immunotools, Cat-No. 21227778) was added. After 2 h of incubation at RT on a shaker, samples were washed before the addition of 3,3′,5,5′-Tetramethylbenzidine. Optical density was read at 450 nm wavelength after 20 min incubation.

### Statistical analysis

Statistical analysis was performed according to the methods indicated in the legends of each figure, using Prism software 6 (GraphPad Software, USA), which was also used for figure design. Statistical significance was defined as p < 0.05.

### Ethics statement

Experimental procedures involving cells from healthy donors were performed with blood samples obtained after written informed consent, and were approved by the Research Ethics Committee of the Oswaldo Cruz Foundation/Fiocruz (Rio de Janeiro, RJ, Brazil) under the number 397-07. Plasma and whole blood samples from HIV-1-infected patients were obtained after written informed consent that was approved by the Human Research Ethics Committee of the General Hospital of Nova Iguaçu, Rio de Janeiro (ID: 008/2010). All experiments were performed in accordance with the relevant guidelines and regulations of both Research Ethics Committee cited above.

## Abbreviations

NETs: Neutrophil extracellular traps
HIV-1: Human immunodeficiency virus type-1
PAMPS: Pathogen-associated molecular patterns
